# Notes of Experiments on Ulcers and Wounds

**Published:** 1855-04

**Authors:** Daniel Brainard

**Affiliations:** Prof. of Surgery in Rush Medical College, Chicago, Surgeon of the U. S. Marine Hospital and of the Mercy Hospital at Chicago, Foreign Corresponding Member of the “Societe de Chirurgie” of Paris, and of the Medical Society of the Canton of Geneva, Switzerland, etc. etc.


THE NORTH-WESTERN

MEDICAL AND SURGICAL JOURNAL.

NEW SERIES.

VOL. IV. APRIL, 1855. NO. 4.

### ORIGINAL COMMUNICATIONS.

ARTICLE I.— *Notes of Experiments on Ulcers and Wounds*, designed to serve as aids to the investigation of the Chemical action which takes place on ulcerated and wounded surfaces. By DANIEL BRAINARD, M D., Prof. of Surgery in Rush Medical College, Chicago, Surgeon of the U. S. Marine Hospital and of the Mercy Hospital at Chicago, Foreign Corresponding Member of the "Societe de Chirurgie" of Paris, and of the Medical Society of the Canton of Geneva, Switzerland, etc. etc.

THERE is no surgical disease concerning which our actual knowledge is less satisfactory than that of ulcers. The definition sometimes given, that an ulcer is a solution of continuity of the soft parts secreting pus, amounts to little less than saying that an ulcer is an ulcer. If we adopt a more recent definition and say that it is "a solution of continuity on which a peculiar action takes place," we do but repeat the former, since all that is known of this action is that it forms pus or some fluid having a greater or less analogy to it.

Still, the latter definition has this great advantage over the former, that it leaves it to be inferred that some action, the nature of which is unknown, does take place on the surface of ulcers, a fact which every observing and reflecting person must have perceived, especially when called upon to explain their pathology.

Since it is known that every moistened animal membrane brought in contact with a gas absorbs it by endosmosis, we might expect that an action of absorption of air would necessarily take place on the surface of ulcers, unless this surface is expressly constituted to prevent this action from taking place. To determine this point was the first object to which my experiments were directed. At the same time an opportunity was offered of ascertaining whether an exhalation of gases takes place from ulcerated surfaces.

### Experiment 1st.

I had the edges of a cupping glass of an oval form, ground so as to fit accurately a surface of ground glass.

This was applied, Nov. 21, 1854, at nine o'clock, A.M., over the surface of a caustic issue, in such a manner as perfectly to prevent the entrance or escape of gas or air. The issue was situated on the fore part of the thigh, immediately below the anterior spinous process of the ilium. It had been formed two weeks previously on account of a rheumatic affection of the hip joint, and at the time of the application of the cup the sides of it presented slight appearances of granulation, but at the bottom the muscular fibres were distinctly visible, the slough having been but recently separated. The issue was near two inches in length, and an inch and a half in breadth.

The cup was removed at 7 o'clock, P.M., after having been on 10 hours.

The cup was found to be much exhausted, the surface of the issue being drawn up as if a part of the air had been pumped out.

The patient stated that it had burned him for two or three hours.

There was on the surface of the ulcer a quantity of gelatinous matter, somewhat like the buffy coat on the surface of inflamed blood. It was about two lines in thickness, and slightly tinged with blood. It gave an alkaline reaction.

The gas remaining in the cup was found to be pure nitrogen. It would not support combustion, and contained no carbonic acid.

### Experiment 2nd.

Nov. 24.—The same glass containing atmospheric air as before was applied at 9 o'clock A.M., and removed at 7 P.M.

The surface of the issue was granulated throughout its whole extent.

On removing the glass the same appearances were noticed as in the first experiment. The gas remaining would not support combustion. Tested for sulphuretted, carboretted and phosphoretted hydrogen, it gave no trace of either. Fluid under the cup gave an alkaline reaction.

### Experiment 3d.

Nov 28.—Cup containing air applied as before at 9 o'clock, A.M. The issue presented on its whole surface healthy granulations, and around its edges the appearance of cicatrization.

Cup removed at 7 o'clock, P.M. Same appearances as in the first and second experiments. The tests of the gas gave the same results as in No. 2.

### Experiment 4th.

Dec. 5.—Cup applied with air as before. Same appearances as in third experiment. Issue covered with healthy granulations nearly level with the surface—cicatrization advancing rapidly.

The fluid found under the cup contained fibrine and exudation corpuscles. Not examined for salts.

### Experiment 5th.

The cup was applied December 8 at 9 o'clock A.M., filled with carbonic acid. It was removed at six o'clock P.M. A vacuum had been produced to a much greater degree than in either of the preceding experiments. Patient complained of pain during the whole time of the application.

Considerable serous fluid and a gelatinous matter was found beneath the cup. Gelatinous matter showed imperfectly formed fibres and exudation corpuscles under the microscope. All the tests of the gas remaining in the cup showed carbonic acid gas.

There were also observed in the fluid dried on the glasses, crystals of the triple phosphate of ammonia and magnesia, and of the chloride of sodium.

### Experiments 6th, 7th and 8th.

Cup applied at 9 A M., containing oxygen gas. Issue cicatrizing rapidly, gave neither acid nor alkaline reaction. Cup removed at 7 P.M. No vacuum had been produced. Fluid on the surface gave acid reaction. Gas in cup gave the reaction of oxygen.

The issue at this time and the granulations rising above the surface was indolent and insensible.

This experiment repeated twice gave the same results. It is thus shown that in certain stages of ulcers the absorption of oxygen ceases.

### Experiments 9th, 10th and 11th.

This issue having been cauterized with the chloride of zinc, and the superficial slough separated and surface granulated, the cup was applied, filled with oxygen, for nine hours. At the end of this time a slight absorption of oxygen had taken place. The surface was hard and insensible. This experiment repeated twice gave the same result.

### Experiment 12th.

Atmospheric air was applied with the cup for nine hours, at the end of which time there was partial absorption of its oxygen.

### Experiment 13th.

Cup containing atmospheric air was applied over a small freshly cut surface, made to form an issue, on the thigh of a patient whose lower members were paralyzed from injury of the spine. At the end of ten hours oxygen was absorbed, and the cup contained carbonic acid.

### Experiments 14th and 15th.

Lint having been pressed into this wound, a cup was applied over it as before. At the end of ten hours the same result was found as in Experiment 13, and ammonia and sulphuretted hydrogen were found mixed with the gases in the cup. This experiment was repeated once with the same result.

These experiments were made in the wards of the Mercy Hospital, at Chicago, during the winter, the atmosphere being at the temperature of about 70° F.

### Conclusions.

We think the results of the foregoing experiments justify us in drawing the following conclusions:

1. Recent ulcers absorb pure oxygen or the oxygen of the atmosphere without giving out carbonic acid.

2. The same ulcers absorb carbonic acid rapidly—this or its combination with soda on the surface may account for this gas not being found in the cup when oxygen has been absorbed.

3. On ulcers absolutely excluded from air, no pus or imperfectly elaborated pus was found.

4. Chloride of sodium is found on the surface of ulcers, to which carbonic acid gas is applied, and from which atmospheric air is excluded.

5. Chronic ulcers do not absorb gases.

6. Fresh wounds absorb oxygen and eliminate carbonic acid, which is not absorbed.

### Remarks.

It will be seen that recent ulcers absorb oxygen and carbonic acid, like dead animal substance, as muscle or membrane, although this action is not attended by the phenomena of putrefaction.

What becomes of the oxygen absorbed, it is impossible without further experiment to say. It is not improbable that in some cases it combines with the fluids on the surface of the ulcer, and forms an irritating compound, which is the cause of unhealthy inflammation. It is known that air was formerly considered to be poisonous to wounds, and although this doctrine has been abandoned the fact that deleterious effects follow the admission of air to large wounds or suppurating surfaces is undeniable.

Oxygen, when absorbed by dead animal matter, combines with a portion of it and forms a ferment which causes putrefaction. In the living body the perpetual change of the fluids prevents putrefaction.

The absorption of oxygen and the elimination of carbonic acid on the surface of wounds, might be regarded as simply an evidence that the action of respirati n takes place there as throughout all the tissues of the body. But it is not probable that this act is so simple. The different effects resulting from wounds left open and those carefully closed, would indicate that some substance is formed on the surface in the former case, which being absorbed causes the constitutional symptoms.

Thsee views are made public at the present time, although in a very imperfect state, in order to attract attention to the subject, and in the hope that they may induce some of the younger members of the profession to take it up, and by a series of careful experiments, determine accurately what are the changes that take place on the surface of the various classes of ulcers. The bearing of the facts thus to be established on questions of physiology and pathology, is it seems to me sufficiently important to repay a careful investigation.

